# Thrombectomy for distal medium vessel occlusion stroke: Combined vs. single-device techniques - A systematic review and meta-analysis

**DOI:** 10.3389/fstro.2023.1126130

**Published:** 2023-01-26

**Authors:** Enver De Wei Loh, Gabriel Yi Ren Kwok, Keith Zhi Xian Toh, Ming Yi Koh, Yao Hao Teo, Yao Neng Teo, Bernard P. L. Chan, Vijay Kumar Sharma, Megan Bi-Jia Ng, Hui Shi Lim, Betsy Soon, Anil Gopinathan, Cunli Yang, Ching-Hui Sia, Pervinder Bhogal, Patrick A. Brouwer, Lukas Meyer, Jens Fiehler, Tommy Andersson, Benjamin Y. Q. Tan, Leonard L. L. Yeo

**Affiliations:** ^1^Lee Kong Chian School of Medicine, Nanyang Technological University, Singapore, Singapore; ^2^Institute of Health Sciences Education, Barts and The London School of Medicine and Dentistry, Queen Mary University of London, London, United Kingdom; ^3^Department of Medicine, Yong Loo Lin School of Medicine, National University of Singapore, Singapore, Singapore; ^4^Division of Neurology, Department of Medicine, National University Hospital, Singapore, Singapore; ^5^Department of Diagnostic Radiology, National University Hospital, Singapore, Singapore; ^6^Division of Interventional Radiology, National University Hospital, Singapore, Singapore; ^7^Department of Diagnostic Radiology, Yong Loo Lin School of Medicine, National University of Singapore, Singapore, Singapore; ^8^Department of Cardiology, National University Heart Centre Singapore, Singapore, Singapore; ^9^Department of Interventional Neuroradiology, The Royal London Hospital, London, United Kingdom; ^10^Cerenovus, Johnson and Johnson MedTech, Irvine, CA, United States; ^11^Department of Diagnostic and Interventional Neuroradiology, University Medical Center Hamburg-Eppendorf, Hamburg, Germany; ^12^Departments of Neuroradiology, Karolinska University Hospital and Clinical Neuroscience, Karolinska Institutet, Stockholm, Sweden; ^13^Department of Medical Imaging, AZ Groeninge Hospital, Kortrijk, Belgium

**Keywords:** distal vessel occlusion, medium vessel occlusion, stent retriever, aspiration, thrombectomy, meta-analysis, acute ischemic stroke

## Abstract

**Background:**

The optimal mechanical thrombectomy technique for acute ischaemic stroke (AIS) caused by distal, medium vessel occlusion (DMVO) is uncertain. We performed a systematic review and meta-analysis evaluating the efficacy and safety of first-line thrombectomy with combined techniques, which entail simultaneous use of a stent retriever and aspiration catheter, vs. single-device techniques, whether stent retriever or direct aspiration alone, for DMVO-AIS patients.

**Methods:**

We systematically searched the PubMed, Embase and Cochrane CENTRAL databases from inception until 2 September 2022 for studies comparing combined and single-device techniques in DMVO-AIS patients. We adopted the Distal Thrombectomy Summit Group's definition of DMVO. Our outcomes were the modified first-pass effect [mFPE; modified Thrombolysis in Cerebral Infarction (mTICI) 2b-3 at first-pass], first-pass effect (FPE; mTICI 2c-3 at first-pass), successful and complete final reperfusion (mTICI 2b-3 and 2c-3 at end of all procedures, respectively), 90-day functional independence (modified Rankin scale 0-2), 90-day mortality, and symptomatic intracranial hemorrhage (sICH).

**Results:**

Nine studies were included, with 477 patients receiving combined techniques, and 670 patients receiving single-device thrombectomy. Combined techniques achieved significantly higher odds of mFPE [odds ratio (OR), 2.12; 95% confidence interval (CI), 1.12–4.02; *p* = 0.021] and FPE (OR, 3.55; 95% CI, 1.97–6.38; *p* < 0.001), with lower odds of sICH (OR, 0.23; 95% CI 0.06–0.93; *p* = 0.040). There were no significant differences in final reperfusion, functional independence (OR, 1.19; 95% CI 0.87–1.63; *p* = 0.658), or mortality (OR, 0.94; 95% CI, 0.50–1.76; *p* = 0.850).

**Conclusions:**

In DMVO-AIS patients, mechanical thrombectomy combining stent retrievers and aspiration catheters achieved higher odds of FPE and lower odds of sICH over single-device techniques. There were no differences in functional independence and mortality. Further trials are warranted to establish these findings.

**Systematic review registration:**

https://www.crd.york.ac.uk/prospero/display_recor d.php?ID=CRD42022370160, identifier: CRD42022370160.

## Introduction

Mechanical thrombectomy has evolved since it was first used for the treatment of large vessel occlusions (LVOs) in acute ischemic stroke (AIS): thrombectomy with stent retriever or direct aspiration is now established as standard-of-care (Class I, Level A evidence) (Powers et al., 2019). More recently, combined techniques, which incorporate simultaneous use of a stent retriever and aspiration catheter, have reported higher reperfusion rates and fewer treatment passes in treating LVO-AIS patients (Yeo et al., 2021; Okuda et al., 2022).

There is, however, comparatively less clarity for AIS patients with distal, medium vessel occlusions (DMVOs), an important cohort contributing significant morbidity worldwide (Saver et al., 2020). The Distal Thrombectomy Summit Group defines DMVOs as occlusions of the anterior cerebral artery, M2-M4 middle cerebral artery (MCA), posterior cerebral artery (PCA), posterior inferior cerebellar artery, anterior inferior cerebellar artery, and/or superior cerebellar artery (Saver et al., 2020). These vessels are longer, narrower, more tortuous, and have thinner arterial walls, thus presenting an increased risk of complications. Since the affected territory can also be smaller, there may be lower margins for benefits in performing mechanical thrombectomy (Saver et al., 2020; Yeo et al., 2021). Given the inherently delicate balance between risk and reperfusion benefits, it is critical that the safest and most effective approach is chosen for first-line thrombectomy.

There has yet to be conclusive evidence for the optimal first-line thrombectomy technique in DMVOs. Randomized-controlled trials have largely focused on anterior circulation LVOs, and the few evaluating M2 AIS patients have largely been inadequately powered (Campbell et al., 2015). The HERMES Collaboration retrospectively pooled individual patient data from seven randomized controlled trials and did show efficacy for mechanical thrombectomy as compared to best medical care in the subgroup of M2 occlusions, but no comparison was made between different thrombectomy techniques (Menon et al., 2019). In the ARISE-II trial, the EmboTrap stent retriever achieved similar rates of good functional outcomes, successful reperfusion and mortality across M1 and M2 occlusions (de Havenon et al., 2021). Some guidelines now cautiously indicate stent retriever thrombectomy can be considered for selected M2 and M3 DMVO-AIS patients (Class IIb, Level C evidence; Powers et al., 2019) although it remains unclear how it compares against other thrombectomy approaches.

As the technical goal of thrombectomy is to achieve reperfusion as early as possible (Powers et al., 2019) the first-pass effect [modified Thrombolysis in Cerebral Infarction (mTICI) scores of 2c-3 after a single thrombectomy device pass] is an independent predictor of favorable long-term outcomes (Zaidat et al., 2018). In the ASTER trial of M2 DMVOs, first-pass effect was achieved in only 45.6% of patients treated with either stent retriever or direct aspiration as first-line strategy (Gory et al., 2018). Meanwhile, the retrospective, multicentre TOPMOST study of posterior circulation DMVOs found that first-line aspiration and stent retriever both achieved similar first-pass effect rates of 53.7 and 44% of patients, respectively (Meyer et al., 2022). These results thus suggest a potential role for combined techniques, given their promising technical efficacy observed in LVO-AIS. We therefore performed a systematic review and meta-analysis of the contemporary literature comparing the efficacy and safety of first-line thrombectomy with combined techniques vs. stent retriever or direct aspiration alone in patients with AIS caused by DMVO. We aimed to elucidate which approach should be considered for first-line thrombectomy in this cohort.

## Methods

A systematic review and meta-analysis was performed in accordance with the Preferred Reporting Items for Systematic Reviews and Meta-Analyses (PRISMA) guidelines. A protocol was established and agreed by all authors before commencing the review, and registered on the International Prospective Register of Systematic Reviews (PROSPERO: registration number CRD42022370160). We sought to analyse all studies that included DMVO-AIS patients and compared combined-device techniques against single-device techniques as the first-line thrombectomy technique.

### Search strategy

We systematically searched the PubMed, Embase and Cochrane CENTRAL databases for observational and randomized-controlled studies comparing combined techniques against single-device thrombectomy from inception to September 2022 (Supplementary Table S1). A manual search of the reference lists of included studies and pertinent review papers was also performed. After removing duplicate records, two authors independently screened all titles and abstracts obtained from the searches, excluding any irrelevant studies. Two authors then independently evaluated full texts of the remaining articles for final inclusion in the review. The review process from electronic searching through the review of full texts was managed on Covidence software, and any disagreements were resolved by discussion.

### Selection criteria

The selection criteria are summarized in Supplementary Table S2. We included all studies that met the following criteria:

1. At least 10 patients with primary or secondary DMVO-AIS, as defined by the Distal Thrombectomy Summit Group consensus statement;
2. At least two treatment arms comparing a combined technique against single-device approach (stent retriever or direct aspiration) as first-line thrombectomy;
3. Provided sufficient data for meta-analysis of at least one technical, clinical or safety outcome.

We evaluated the following technical outcomes:

1. Modified first-pass effect (mFPE), defined as mTICI 2b-3 at the end of first pass procedure;
2. First-pass effect (FPE), defined as mTICI 2c-3 at the end of first pass procedure (Zaidat et al., 2018)
3. Successful final reperfusion, defined as mTICI 2b-3 at the end of all procedures;
4. Complete final reperfusion, defined as mTICI 2c-3 at the end of all procedures.

We also evaluated the following clinical and safety outcomes:

1. Functional independence, defined as a 90-day modified Rankin Scale (mRS) score of 0-2;
2. 90-day mortality;
3. Incidence of symptomatic intracranial hemorrhage (sICH).

### Data extraction and quality assessment

Two authors independently extracted study data using a standardized data collection form. We extracted data pertaining to the abovementioned outcomes, as well as the following study and patient characteristics: study year, design, cohort size, age, sex, presence of established risk factors, underlying medical conditions, occlusion sites, National Institutes of Health Stroke Scale (NIHSS) scores on admission, onset to groin puncture times, number of passes and procedure times.

We used the Newcastle-Ottawa Scale (NOS) to assess cohort and case-control studies. The NOS evaluates the selection of study groups, comparability of the groups, and the comparability of the outcomes. Each study was independently graded by two authors as having a high (< 5 score), moderate (5-7 score) or low (8-9 score) risk of bias, with disagreement resolved by discussion and consensus.

### Statistical analyses

We calculated the pooled odds ratio (OR) and associated 95% confidence interval (CI) for each outcome, using a random effects model to account for expected clinical heterogeneity in thrombectomy techniques and devices used in the studies. Outcomes were compared between combined techniques and any single-device techniques, stent retriever alone, and direct aspiration alone. We also calculated prediction intervals, which provide a range of effect sizes expected in any future studies. Variance between individual studies was estimated via the method of restricted maximum-likelihood. Heterogeneity was assessed using the Cochran's Q test and quantified with the I^2^ statistic; ranges of 0–30%, 31–60%, and >60% indicated low, moderate, and substantial heterogeneity, respectively. We performed subgroup analysis by occlusion site, but not for other variables such as age, sex, and stroke etiology because the relevant data was not available. Funnel plot analysis for publication bias and small-studies effects was not possible as less than 10 studies were included in a single meta-analysis. All statistical analyses were performed on RStudio version 4.2.1 using methods and plots provided by the *meta* package (Balduzzi et al., 2019).

## Results

**Figure 1:**
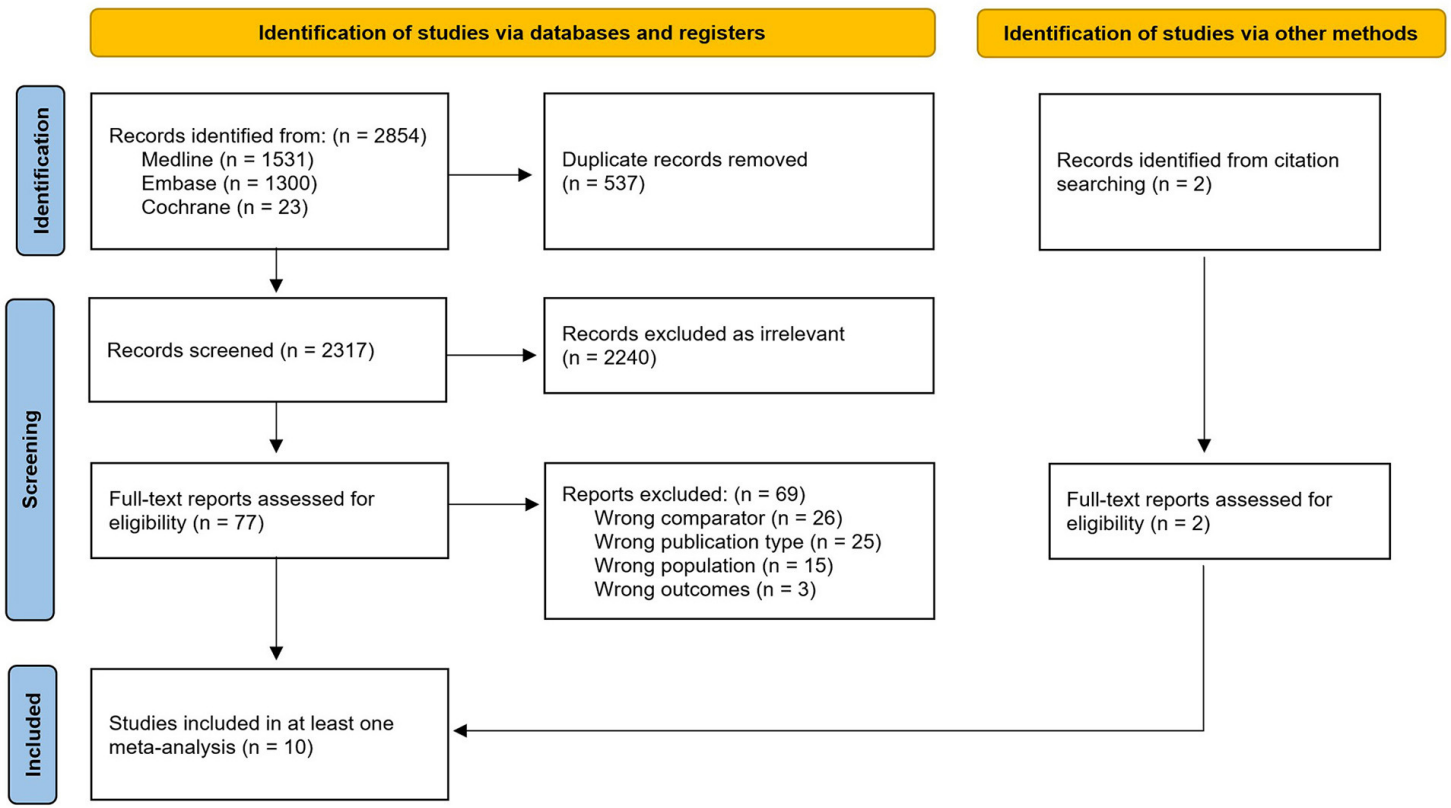
PRISMA flow diagram outlining the search and screening process.

The systematic search yielded 2,854 records, from which 537 duplicates were removed. We excluded a further 2,240 articles based on the initial screening of titles and abstracts, before identifying eight full texts for inclusion. Two additional papers were identified through manual handsearching. Finally, a total of 10 papers reporting the results of nine studies were included in at least one meta-analysis (Figure 1).

### Study characteristics

A total of 1,147 patients were included across 9 studies, all retrospective cohort studies. Most of these studies had low risks of bias. The characteristics of the included studies are summarized in Table 1.

**Table 1:** Characteristics of included studies.

	**Brehm et al. (2019)**	**Miura et al. (2019)**	**Pérez-García et al. (2020)**	**Haussen et al. (2020)**	**Renieri et al. (2021)**	**Meyer et al. (2022)**	**Okuda et al. (2022)**	**Baig et al. (2022)**	**Farouki et al. (2022), Hulscher et al. (2022)**
**Country**	Germany	Japan	Spain	USA	North America and Europe	International	Japan	USA	Belgium
**Design**	Retrospective cohort	Retrospective cohort	Retrospective cohort	Retrospective cohort	Retrospective cohort	Retrospective cohort	Retrospective cohort	Retrospective cohort	Retrospective cohort
**Study period**	January 2014–September 2017	January 2016–December 2018	February 2017–January 2020	January 2014–July 2018	January 2017–May 2020	January 2014–June 2020	January 2013–January 2020	January 2015–July 2020	January 2018– January 2021
**Occlusion site(s)**	M2	M2-3, A2-3	M2-3, A1-3, P1-3	M2-3, A1-3, P1-2	M2	P2-3	M2	P1-2	A2, P1, M2-3
**Number treated**	22	65	102	159	465	141	111	21	61^&^
**NOS, total (S,C,E)**	8 (3 ,2, 3)	9 (4, 2, 3)	7 (2, 2, 3)	7 (3, 2, 2)	9 (4, 2, 3)	8 (4, 2, 2)	9 (4, 2, 3)	8 (3, 2, 3)	9 (4, 2, 3)
**Allocation method**	Physician discretion	Physician discretion	Time period^@^	Not specified	Physician discretion	Not specified	Physician discretion	Not specified	Physician discretion
**Combined techniques**									
**Technique**	SAVE	DCT with BGC	BEMP with BGC	BEMP ± BGC (*n =* 23, 92%)^**^	Combined	Combined	SCT ± BGC (*n =* 50, 98.4%)	SRA	Combined
**Number treated**	12	28	53	22	239	38	51	7	27
**IVT use, n (%)**	(68.7)^*^	9 (32)	22 (41.5)	5 (23)	120 (50.2)	–	(45.8)^*^	4 (57.14)	–
**Age, years**	74.5 ± 11.45^*^	79 ± 10	67.7 ± 13.7	67 (59-76)	71.11 ± 14.56	–	81 (70.2-86)^*^	58 (55-70.5)	–
**Onset to puncture, mins**	–	–	258 ± 198	462 (186-666)	262 (180-453)	–	185 (123.5-370)^*^	197 (186-205)	–
**NIHSS**	16 (12-20)^*^	13 (9-19)	16 (8.75-20.25)	17 (9-23)	–	–	20 (14-24)^*^	8 (4-10.5)	–
**ASPECTS**	8 (7-9)^*^	8 (7-9)	9 (8-10)	8 (6-10)	–	–	9 (7-10)^*^	–	–
**Number of passes**	-	1 (1-2)	1(1-1)	1 (1-1)	1.7 ± 1.0	–	1.72 ± 0.92^*^	2 (1-2.5)	–
**Groin to reperfusion time, mins**	51.8 ± 29.6	31 (24-43)	44.9 ± 21.6	–	–	–	43 (31.5-69)^*^	–	–
**Devices**	8F Mach1, 8F Vista Brite Tip, NeuronMAX 088, Trevo XP ProVue Retriever, pRESET, APERIO, 3D Septembererator	3MAX, 4MAX, Solitaire 2, Trevo XP ProVue, Trevo Pro 14 Stryker, Trevo XP ProVue	Arc Mini, 3MAX	Trevo Stryker, Trevo 14, 3MAX	–	—	5MAX ACE, ACE 68, 5MAX, CATALYST 6, SOFIA 5F, SOFIA 6F, 4MAX, 3MAX, Trevo XP ProVue, Solitaire	–	–
**Technique**	ADAPT	ADAPT (*n =* 29) or SR (*n =* 8), with BGC	Mini SR with BGC	Standard techniques ± BGC (*n =* 134, 93%)	SR (*n =* 133) or DA (*n =* 93)	SR (*n =* 62) or DA (*n =* 41)	SR (*n =* 17) or DA (*n =* 43) ± BGC (*n =* 58, 96.6%)	ADAPT (*n =* 9) or SR (*n =* 5)	SR (*n =* 14) or DA (*n =* 19)
**Number treated**	10	37	49	137	226	103	60	14	34^&^
**IVT use, n (%)**	(69.4)^*^	20 (54)	19 (38.8)	57 (41)	111 (49.1)	–	(49.1)^*^	6 (42.86)	–
**Age, years**	72.6 ± 14.1^*^	76 ± 12	68.7 ± 16.2	66 (55-74)	SR: 71.73 ±15.16 DA: 72.07 ±12.34	–	79 (70-84)^*^	73.5 (69-81.5)	–
**Onset to puncture, mins**	–	–	270 ± 132	390 (228-726)	SR: 195.5 (150-283.5) DA: 262 (194-385)	–	196.5 (134.2-286)^*^	173.5 (112.5-240.25)	–
**NIHSS**	16 (9-20)^*^	15 (7-19)	16.5 (12-20.12)	16 (11-22)	-	–	18 (14-23)^*^	9 (7-20.75)	–
**ASPECTS**	8 (7-9)^*^	8 (7-9)	8.5 (7-10)	9 (7-10)	–	–	10 (8-10)^*^	–	–
**Number of passes**	–	2 (2-3)	1 (1-2)	1 (1-2)	–	–	1.99 ± 1.0^*^	2 (1-2)	—
**Groin to reperfusion time, min**	49.4 ± 26.2	43 (34-68)	55.4 ± 30.9	–	–	–	55 (38-82.2)	–	–
**Devices**	8F Mach1, 8F Vista Brite Tip, NeuronMAX 088, SOFIA 6F, SOFIA plus, SOFIA 5F, CATALYST 6, 5MAX ACE, ACE 68, 5MAX, 4 MAX, CATALYST 5, ACE 64	3MAX, 4MAX, 5MAX, ACE60, ACE68, Trevo XP ProVue, Solitaire 2	Catch Mini 3x15mm, Catch Mini 3x20mm, Aperio 3.5x28mm	3 mm Trevo, 3MAX	–	–	5MAX, 5MAX ACE, 4MAX, ACE 68, Trevo XP ProVue, Solitaire	–	–

Values presented are mean ± standard deviation, or median (interquartile range). NOS, Newcastle-Ottawa Scale; S,C,E, selection, comparability, outcome; NIHSS, National Institutes of Health Stroke Scale; ASPECTS, Alberta Stroke Program Early CT Score; DCT, distal combined technique; BEMP, blind exchange/mini pinning technique; BGC, balloon-guide catheter; SAVE, stent-retriever assisted vacuum-locked extraction; SCT, single-unit combined technique; SRA, stent retriever-assisted aspiration; SR, stent retriever; DA, direct aspiration; ADAPT, a direct aspiration first-pass technique. ^*^Data not available for the DMVO-AIS subgroup of patients; percentages refer to the overall study population, which included anterior LVOs. ^**^25 vessels from 22 patients were treated in this arm, of which 23 vessels incorporated BGC. ^@^Mini stent retriever was first-line in the earlier study period, when low-profile aspiration catheters had not yet been released. In the later study period, combined techniques became the first-line technique. ^&^Farouki et al. (2022) reported data for 60 patients (27 combined technique, 33 single-device technique), omitting one patient from the single-device arm due to missing data.

Where it was possible to pool patient characteristics, the study population comprised 409/820 (49.9%) males (Table 2). Most studies also reported established risk factors for stroke such as hypertension (340/488, 69.7%), dyslipidaemia (176/488, 36.1%), diabetes mellitus (105/488, 21.5%), atrial fibrillation (212/488, 43.4%), and smoking (127/434, 29.3%). Patient characteristics of individual studies are provided in Supplementary Table S3.

**Table 2:** Pooled patient characteristics from included studies.

	**Male (%)**	**Hypertension (%)**	**Dyslipidemia (%)**	**Diabetes mellitus (%)**	**Atrial fibrillation (%)**	**Smoking (%)**
Combined techniques	220/449 (49.0)	155/210 (73.8)	79/210 (37.6)	42/210 (20)	99/210 (47.1)	86/292 (29.5)
Single-device techniques	189/371 (50.9)	185/278 (66.5)	97/278 (34.9)	63/278 (22.7)	113/278 (40.6)	41/142 (28.9)
Total	409/820 (49.9)	340/488 (69.7)	176/488 (36.1)	105/488 (21.5)	212/488 (43.4)	127/434 (29.3)

Overall, 477 patients received first-line thrombectomy via combined techniques, while 670 patients underwent stent retriever or direct aspiration alone as their primary treatment. This included 288 patients who received stent retriever alone and 244 patients who received direct aspiration alone, although the breakdown of the single-device arm was not specified in some studies. One study included 137 patients with 144 occluded arteries in the single-device arm, but only specified the number of vessels treated by each technique (*n* = 92 stent retriever, *n* = 52 direct aspiration; Haussen et al., 2020). Four studies (Miura et al., 2019; Haussen et al., 2020; Pérez-García et al., 2020; Okuda et al., 2022) used balloon-guide catheters (BGC) for all or nearly-all procedures in the same proportions across both arms, but none compared BGC against non-BGC procedural techniques.

### Technical outcomes

**Figure 2:**
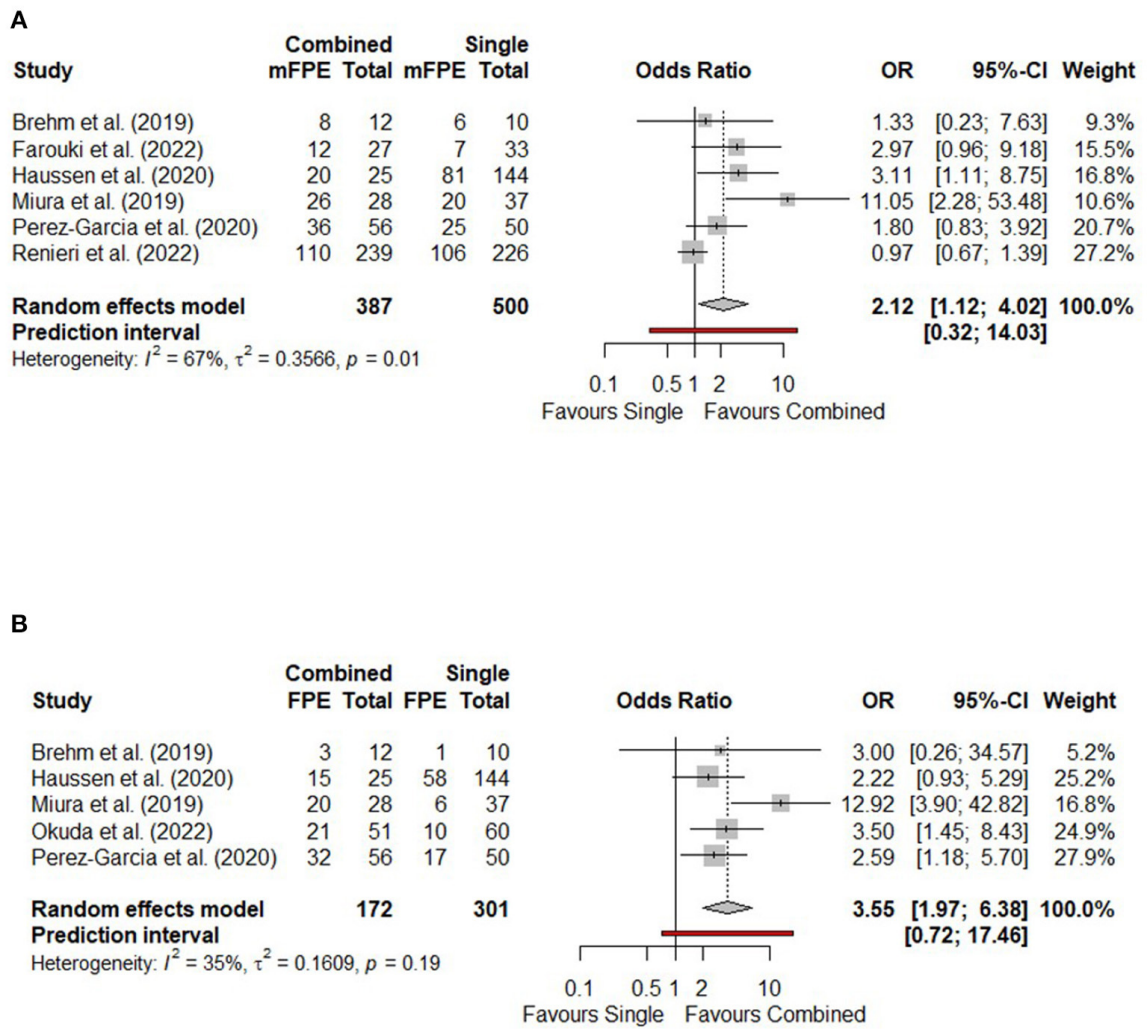
Forest plots comparing technical outcomes of combined vs. single-device techniques in terms of **(A)** Modified first pass effect (mTICI 2b-3); **(B)** First pass effect (mTICI 2c-3). CI, confidence interval; FPE, first pass effect; mFPE, modified first pass effect; mTICI, modified Thrombolysis in Cerebral Infarction; OR, odds ratio.

Six studies incorporating 887 patients were included in the analysis of the mFPE (2b-3). Combined techniques produced higher odds of the mFPE than single-device techniques, with substantial heterogeneity (OR 2.12, 95% CI 1.12–4.02, *p* = 0.021, I^2^ = 67%) (Figure 2A). There were no significant differences in odds of the mFPE between combined techniques and stent retriever alone (Supplementary Figure S1A), or direct aspiration alone (Supplementary Figure S2A).

Five studies reported the FPE (2c-3), comprising 473 patients. One study (Pérez-García et al., 2020) used the expanded Thrombolysis in Cerebral Ischemia (eTICI) scale instead of mTICI; for the purposes of meta-analysis, eTICI 2b/2c/3 were considered equivalent to mTICI 2b/2c/3 respectively. Combined techniques achieved higher odds of the FPE than single-device techniques, with moderate heterogeneity (OR 3.55, 95% CI 1.97–6.38, *p* < 0.001, I^2^ = 35%) (Figure 2B). One study compared a combined technique and stent retriever alone, with the combined technique yielding higher odds of the FPE (OR 2.58, 95% CI 1.18–5.70) (Pérez-García et al., 2020). Another study compared a combined technique and direct aspiration alone, with similar odds of the FPE (OR 3.00, 95% CI 0.26-34.6) (Brehm et al., 2019).

Seven studies incorporating 989 patients were included in the analysis of successful (2b-3) and complete (2c-3) final reperfusion. There were no significant differences between combined and single-device techniques, with moderate heterogeneity, in terms of successful or complete final reperfusion (Supplementary Figure S3). There were also no significant differences in final reperfusion between combined techniques and stent retriever alone (Supplementary Figures S1B, C), or direct aspiration alone (Supplementary Figures S2B, C).

### Clinical and safety outcomes

**Figure 3:**
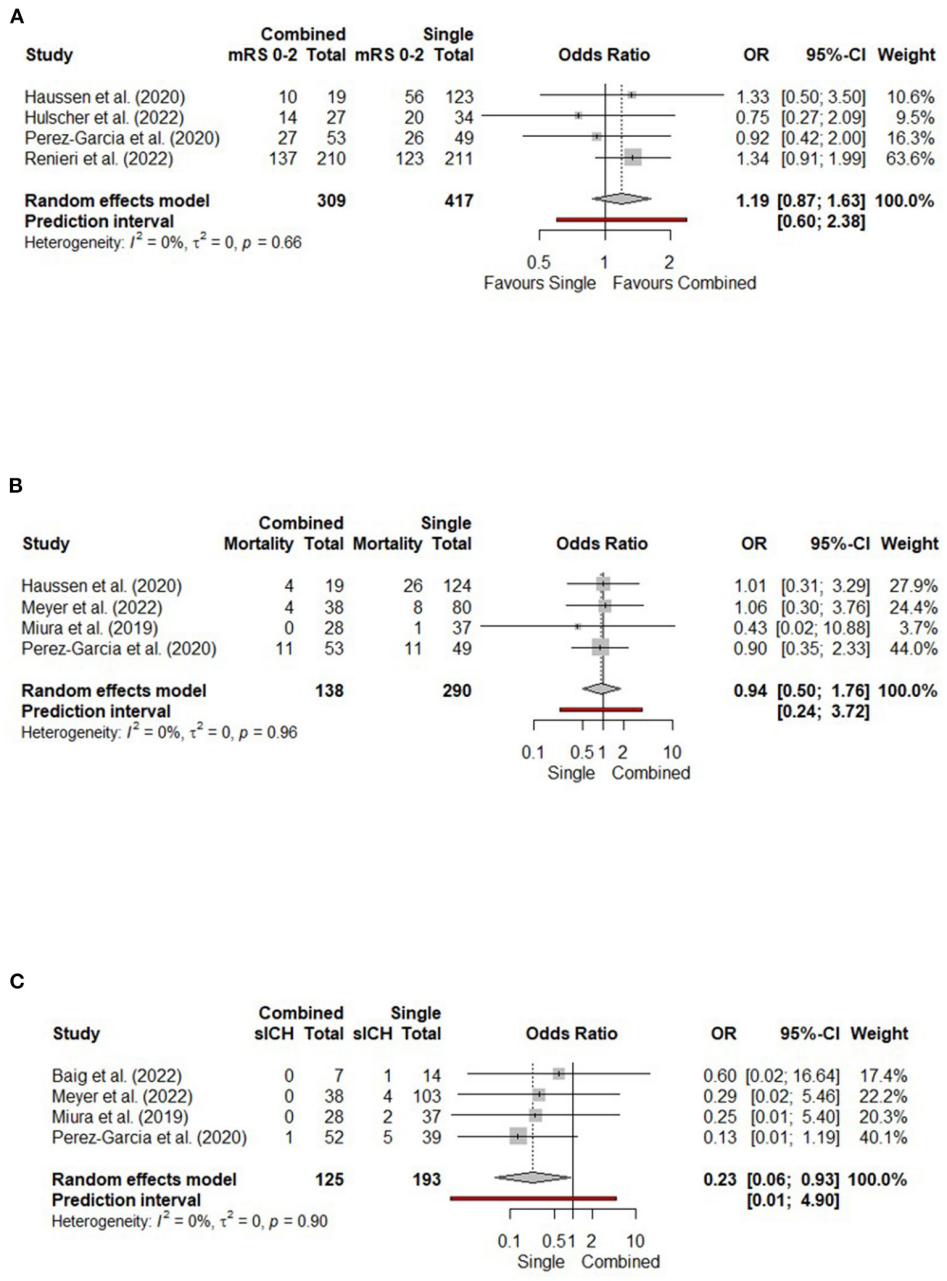
Forest plots comparing clinical and safety outcomes of combined vs. single-device techniques in terms of **(A)** Functional independence (90-day mRS 0-2); **(B)** 90-day mortality; **(C)** Symptomatic intracranial hemorrhage (sICH). CI, confidence interval; mRS, modified Rankin scale; OR, odds ratio.

Four studies incorporating 726 patients were included in the analysis of functional independence at 90 days. There were no significant differences between combined and single-device techniques in 90-day functional independence, with low heterogeneity (OR 1.19, 95% CI 0.87–1.63, *p* = 0.658, I^2^ = 0%) (Figure 3A). There were also no significant differences between combined techniques and stent retriever alone (Supplementary Figure S1D), or direct aspiration alone (Supplementary Figure S2D).

Four studies incorporating 428 patients were included in the analysis of 90-day mortality. There were no significant differences between combined and single-device thrombectomy, with low heterogeneity (OR 0.94, 95% CI 0.50–1.76, *p* = 0.850, I^2^ = 0%) (Figure 3B). There were no significant differences between combined techniques and stent retriever alone (Supplementary Figure S1E). One study compared combined techniques with direct aspiration only, finding no significant differences in overall mortality (OR 0.78, 95% CI 0.19–3.15) (Meyer et al., 2022).

Four studies incorporating 318 patients were included in the analysis of sICH (Figure 3C). Combined techniques yielded lower odds of sICH compared to single-device approaches, with low heterogeneity (OR 0.23, 95% CI 0.06–0.93, *p* = 0.040, I^2^ = 0%). In two studies, combined techniques produced lower odds of sICH compared to stent retriever alone (OR 0.16, 95% CI 0.03–0.93, *p* = 0.041; Supplementary Figure S1F). In two studies comparing combined techniques and direct aspiration alone, there was no significant difference in odds of sICH (Supplementary Figure S2E).

### Subgroup analysis of technical outcomes by occlusion site

Among the nine included studies, three examined M2 occlusions only, two included PCA occlusions only and four included occlusions in any distal medium vessels (DMVO subgroup). In M2 occlusions, combined techniques yielded higher odds of FPE compared to single-device approaches (OR 3.44, 95% CI 1.50–7.86), but there were no significant differences in odds of mFPE, successful final reperfusion and complete final reperfusion (Supplementary Figure S4). In PCA occlusions, combined and single-device approaches achieved similar odds of successful or complete final reperfusion. In the DMVO subgroup, combined techniques achieved higher odds of mFPE (OR 2.91, 95% CI 1.62–5.23, I^2^ = 29%), FPE (OR 3.87, 95% CI 1.41–10.63, I^2^ = 67%) and complete final reperfusion (OR 2.37, 95% CI 1.30–4.30, I^2^ = 32%), but similar odds of successful fina l reperfusion (Supplementary Figure S4). Only subgroup differences for mFPE were significant between M2 and DMVOs (*p* < 0.01), with no overlap in their 95% confidence intervals.

## Discussion

This systematic review and meta-analysis of 1,147 patients from 9 studies suggests that first-line combined techniques for DMVOs are safer and achieve better technical results than stent retriever or aspiration thrombectomy alone, with similar clinical outcomes. Compared to single-device approaches, combined techniques achieved: higher odds of successful and/or complete reperfusion at first-pass and lower odds of sICH, although this did not translate to improved 90-day functional independence and mortality. To the best of our knowledge, our study represents the first systematic review and meta-analysis comparing combined techniques against traditional single-device thrombectomy approaches in this particular subgroup of patients.

Our study indicates that using a stent retriever together with contact aspiration is 2–3 times more likely to achieve successful or complete first-pass reperfusion than either device alone, thus demonstrating improved technical efficacy. Although successful reperfusion has traditionally been defined as mTICI 2b-3 (Powers et al., 2019), a higher angiographic score of mTICI 2c-3 has been associated with even better functional outcomes and lower mortality rates, and has been proposed as the new benchmark for a successful mechanical thrombectomy (Dargazanli et al., 2018; Rizvi et al., 2019). Combined techniques included blind exchange with mini-pinning (BEMP) (Haussen et al., 2020; Pérez-García et al., 2020), the single-unit combined technique (Okuda et al., 2022), and the stent-retriever assisted vacuum-locked extraction (SAVE) technique (Brehm et al., 2019). With two devices in the occluded vessel, thromboembolic clots can be securely captured from both sides via a stent retriever inserted distally and an aspiration catheter placed proximally, thus enhancing clot removal (Massari et al., 2016; McTaggart et al., 2017; Maus et al., 2018). A “pinning technique” can be adopted, where a stent retriever is deployed through an intermediate catheter with added local aspiration. This ensures the vector of force is transmitted more effectively during retraction of the devices, minimizing deformation of the tortuous distal vessels (Yoo and Andersson, 2017). Thus, multi-device approaches provide a critical advantage in DMVOs, by minimizing the higher risks of clot fragmentation and longer device-withdrawal risks for subarachnoid hemorrhage (SAH) caused by vessel size and tortuosity (Haussen et al., 2020; Pérez-García et al., 2020). Conversely, many aspiration catheters may not fit into distal vasculature, creating a bias toward larger vessels being treated by combination techniques and yielding concomitantly better technical outcomes. However, subgroup analysis revealed that differences in technical outcomes between the larger M2 vessels and other DMVOs were only observed in mFPE, where combined techniques improved odds of mFPE in all DMVOs but not when limited to M2 occlusions only. Unfortunately, vessel sizes were not specified by the study authors, so we were unable to perform further analysis.

We found that combined and single-device approaches ultimately achieved similar odds of successful and complete final reperfusion after further attempts and rescue maneuvers. There were also mixed results among the included studies on the procedural characteristics of combined techniques: three studies found that combined techniques required fewer passes, lower need for rescue therapy, and achieved shorter puncture-to-recanalization times (Miura et al., 2019; Pérez-García et al., 2020; Okuda et al., 2022) although other studies did not report any significant differences (Brehm et al., 2019; Haussen et al., 2020; Renieri et al., 2021). Our meta-analysis supports the descriptive findings from a recent systematic review of first-line thrombectomy strategy for DMVOs, which reported that combined techniques may produce better recanalization and functional outcomes than stent retriever or aspiration alone (Bilgin et al., 2022). In contrast to the aforementioned systematic review without a meta-analysis by Bilgin et al. (2022), our study examined a narrower clinical question focused solely on combined vs. single-device techniques, with low to moderate heterogeneity among most included studies. Hence, while combined techniques may achieve greater technical efficacy than either device alone, they also reflect the intrinsic limits of the current generation of thrombectomy devices. Since thrombectomy devices suited for distal, medium vessels are relatively new, continued advances in the technology hold promise for better technical outcomes in the future.

Hemorrhage is a feared complication of thrombectomy, especially in DMVOs where the thinner arterial walls cause greater risk of vessel perforation (Saver et al., 2020). However, our study found that despite involving more devices, combined techniques lower the odds of sICH compared with single-device approaches, particularly stent retrievers. In terms of intraprocedural complications of SAH, three studies found no significant difference between combined and single-device techniques (Miura et al., 2019; Haussen et al., 2020; Pérez-García et al., 2020). However, one study observed that the combined technique led to an increased risk of SAH than direct aspiration (OR 4.6, 95% CI 1.1–20.9) (Renieri et al., 2021). Three studies reported no difference in intraparenchymal hemorrhage rates (Haussen et al., 2020; Pérez-García et al., 2020; Renieri et al., 2021). The reduced haemorrhagic risk can be partly explained by the possible need for fewer thrombectomy passes that each carry concomitant risk of arterial perforation (Mokin et al., 2017). In some techniques, the use of one device can also ameliorate risks created by the other. For instance, the BEMP technique reduces the risk of vessel laceration by capturing the proximal part of the mini stent retriever within the low-profile aspiration catheter, hence there is less retriever exposed and in contact with the arterial wall, reducing the radial and tractional force exerted by the mini stent retriever on the vessel walls (Haussen et al., 2020; Pérez-García et al., 2020). Other procedural complications were also comparable between combined and single-device approaches: Haussen et al. (2020) reported no significant difference in rates of arterial spasm. Miura et al. (2019) found no significant difference in procedure-related adverse events. Meyer et al. (2022) made no comparison to a first-line combined approach, but observed slightly higher periprocedural complication rates of downstream embolisation, embolisation to new territory and iatrogenic vessel injury in posterior circulation DMVO treated with first-pass stent retriever than first-pass aspiration.

In our meta-analysis, the technical efficacy of combined techniques did not translate into better clinical outcomes, with similar functional independence and mortality at 90 days. This contrasts with previous studies identifying first-pass reperfusion as an independent predictor of better clinical outcomes (Zaidat et al., 2018; García-Tornel et al., 2019). However, DMVOs were under-represented in these studies, so the significance of the FPE has yet to be well-evaluated in DMVOs. Furthermore, DMVOs encompass a diversity of clinical syndromes that differ by occlusion location and thus result in varying functional compromise, even after endovascular therapy (Saver et al., 2020). Within M2 occlusions, it has also been observed that anatomical characteristics of the occlusions lend themselves differently to endovascular thrombectomy and may thereby affect clinical outcomes (Menon et al., 2019). It is therefore possible that the FPE has less predictive value in DMVOs, although it is worth noting that we were unable to adjust for potential confounders such as patient age, onset-to-groin time, and NIHSS score at baseline in our meta-analysis. Given the non-randomized allocation of patients, there is potential for selection biases. Finally, because our intention-to-treat analysis examined clinical and safety outcomes after all thrombectomy passes and rescue procedures had been completed, without distinguishing the patients who had achieved the FPE, these metrics may also underestimate the predictive value of the FPE. These conjectures should be interpreted with caution, since our analyses were neither appropriately designed nor adequately powered to test them. Nonetheless, there appears to be clinical equipoise between combined and single-device techniques in terms of functional outcomes and overall mortality, calling into question whether these long-term outcomes justify the expense of using an additional thrombectomy device in combined techniques. Further studies are warranted into the association of FPE with clinical outcomes in DMVO-AIS, while investigations into the cost-effectiveness of combined techniques, and in which specific subgroups of patients, may better inform the use of these techniques.

Our study has several important limitations. Firstly, all included studies are of retrospective, observational design, with patients allocated to different therapies based on physician discretion in most studies, and thus its inherent risks of selection bias. The chosen thrombectomy technique may be influenced by angiographic findings such as size of lumen, distal location and tortuosity of vessel which limit the feasibility of combined techniques, hence a patient for whom combined techniques is considered may not represent the typical DMVO patient. However, most of the included observational studies did not adjust for any confounding variables. Future prospective studies will be important to validate our findings. Secondly, there was significant clinical heterogeneity within both the combined and single-device treatment arms. Patients in the single-device arm may have received either stent retriever or direct aspiration as their first-line management, while the combined techniques assessed in our study encompassed a variety of different techniques. To minimize these, we performed multiple meta-analyses comparing combined techniques with any single-device technique, stent retrievers alone, and direct aspiration alone. However, as the latter two analyses incorporated very few studies, it was not possible to obtain precise effects estimates.

## Conclusions

In DMVO-AIS patients, mechanical thrombectomy techniques combining stent retrievers and aspiration catheters achieved higher odds of FPE and lower odds of sICH over single-device techniques. There were no differences in functional independence and mortality. Further trials should be considered to substantiate the efficacy and safety of these combined thrombectomy techniques.

## Data availability statement

The original contributions presented in the study are included in the article/Supplementary material, further inquiries can be directed to the corresponding author.

## Author contributions

EL, GK, KT, and MK were responsible for literature search, risk of bias assessment, and data extraction. EL and GK wrote the first draft of the manuscript. BT and LY contributed equally to conception, design, and supervision of the study. All authors contributed to critical revision of the manuscript for intellectual content, have read, and approved the final submitted manuscript.
