# Supplementary material for: Thrombectomy for distal medium vessel occlusion stroke: Combined vs. single-device techniques - A systematic review and meta-analysis
Source: Front Stroke. 2023 Jan 26;2:1126130. doi: 10.3389/fstro.2023.1126130 (PMC12802724; doi:10.3389/fstro.2023.1126130)
Supplement: Supplementary file 1 [file Data_Sheet_1.pdf]

Supplemental Material

Table S1. Search strategies for PubMed, Embase and Cochrane CENTRAL databases.

Table S2. PICOS outline of the study selection criteria.

Table S3. Clinical characteristics of included patients.

Figure S1. Forest plots comparing outcomes of combined techniques versus stent retriever alone in terms of: (A) mFPE (mTICI 2b-3); (B) successful final reperfusion (mTICI 2b-3); (C) complete final reperfusion (mTICI 2c-3); (D) functional independence (90-day mRS 0-2); (E) 90-day mortality; (F) symptomatic intracranial hemorrhage (sICH).

Figure S2. Forest plots comparing outcomes of combined techniques versus direct aspiration alone in terms of: (A) mFPE (mTICI 2b-3); (B) successful final reperfusion (mTICI 2b-3); (C) complete final reperfusion (mTICI 2c-3); (D) functional independence (90-day mRS 0-2); (E) symptomatic intracranial hemorrhage (sICH).

Figure S3. Forest plots comparing technical outcomes of combined versus single-device techniques in terms of: (A) successful final reperfusion (mTICI 2b-3); (B) complete final reperfusion (mTICI 2c-3).

Figure S4. Forest plots comparing technical outcomes of combined versus single-device techniques with subgroup analysis by occlusion site, in terms of: (A) modified first-pass effect (mFPE); (B) first-pass effect (FPE); (C) successful final reperfusion (mTICI 2b-3); (D) complete final reperfusion (mTICI 2c-3)

Table S1. Search strategies for PubMed, Embase and Cochrane CENTRAL databases.

**PubMed Search Strategy**	
#1	ischemic stroke OR ischaemic stroke
#2	"distal vessel" OR "medium vessel" OR "DMVO" OR "MEVO" OR "ACA" OR "anterior cerebral artery" OR "PCA" OR "posterior cerebral artery" OR "MCA" OR "middle cerebral artery" OR "PICA" OR "posterior inferior cerebellar artery" OR "AICA" OR "anterior inferior cerebellar artery" OR "SCA" OR "superior cerebellar artery" OR "posterior circulation" OR M2 OR M3 OR M4 OR M5 OR P1 OR P2 OR P3 OR A1 OR A2 OR A3
#3	Thrombectomy OR Embolectomy
#4	Aspiration thrombectomy OR Percutaneous aspiration thrombectomy OR Stent retriever OR Stentriever OR ADAPT OR "A direct aspiration, first pass technique" OR Direct thrombus aspiration OR Direct aspiration OR Stents OR catheter OR microcatheter OR neuroangiography OR Medtronic Solitaire OR Medtronic Mindframe Capture OR Balt Catch OR Gateway OR Penumbra OR Trevo ProVue OR Trevo XP OR Covidien Solitaire OR Revive SE OR Concentric Merci OR Penumbra system
#5	#1 AND #2 AND #3 AND #4

**Embase Search Strategy**	
#1	ischemic stroke OR ischaemic stroke
#2	"distal vessel" OR "medium vessel" OR "DMVO" OR "MEVO" OR "ACA" OR "anterior cerebral artery" OR "PCA" OR "posterior cerebral artery" OR "MCA" OR "middle cerebral artery" OR "PICA" OR "posterior inferior cerebellar artery" OR "AICA" OR "anterior inferior cerebellar artery" OR "SCA" OR "superior cerebellar artery" OR "posterior circulation" OR M2 OR M3 OR M4 OR M5 OR P1 OR P2 OR P3 OR A1 OR A2 OR A3
#3	Thrombectomy OR Embolectomy
#4	Aspiration thrombectomy OR Percutaneous aspiration thrombectomy OR Stent retriever OR Stentriever OR ADAPT OR "A direct aspiration, first pass technique" OR Direct thrombus aspiration OR Direct aspiration OR Stents OR catheter OR microcatheter OR neuroangiography OR Medtronic Solitaire OR Medtronic Mindframe Capture OR Balt Catch OR Gateway OR Penumbra OR Trevo ProVue OR Trevo XP OR Covidien Solitaire OR Revive SE OR Concentric Merci OR Penumbra system
#5	#1 AND #2 AND #3 AND #4

**Cochrane CENTRAL Search Strategy**	
#1	ischemic stroke OR ischaemic stroke
#2	"distal vessel" OR "medium vessel" OR "DMVO" OR "MEVO" OR "ACA" OR "anterior cerebral artery" OR "PCA" OR "posterior cerebral artery" OR "MCA" OR "middle cerebral artery" OR "PICA" OR "posterior inferior cerebellar artery" OR "AICA" OR "anterior inferior cerebellar artery" OR "SCA" OR "superior cerebellar artery" OR "posterior circulation" OR M2 OR M3 OR M4 OR M5 OR P1 OR P2 OR P3 OR A1 OR A2 OR A3
#3	Thrombectomy OR Embolectomy
#4	Aspiration thrombectomy OR Percutaneous aspiration thrombectomy OR Stent retriever OR Stentriever OR ADAPT OR "A direct aspiration, first pass technique" OR Direct thrombus aspiration OR Direct aspiration OR Stents OR catheter OR microcatheter OR neuroangiography OR Medtronic Solitaire OR Medtronic Mindframe Capture OR Balt Catch OR Gateway OR Penumbra OR Trevo ProVue OR Trevo XP OR Covidien Solitaire OR Revive SE OR Concentric Merci OR Penumbra system
#5	#1 AND #2 AND #3 AND #4

Table S2. PICOS outline of the study selection criteria.

	**Inclusion Criteria**
**Population**	Patients with primary or secondary DMVO-AIS.
**Intervention**	Combined technique as first-line thrombectomy.
**Comparator**	Stent retriever or direct aspiration only as first-line thrombectomy technique.
**Outcomes**	*Technical outcomes*<br>- Modified first-pass effect (mFPE), defined as mTICI 2b-3 at the end of first pass procedure.<br>- First-pass effect (FPE), defined as mTICI 2c-3 at the end of first pass procedure.<br>- Successful final reperfusion, defined as mTICI 2b-3 at the end of all procedures.<br>- Complete final reperfusion, defined as mTICI 2c-3 at the end of all procedures.<br><br>*Clinical & safety outcomes*<br>- Functional independence, defined as a 90-day modified Rankin Scale (mRS) score of 0-2<br>- 90-day mortality<br>- Incidence of symptomatic intracranial hemorrhage (sICH)
**Study Design**	- Case-control studies, cohort studies, randomized-controlled trials.<br>- At least 10 patients across both arms.<br>- English language.

Table S3. Clinical characteristics of included patients.

	Brehm et al. (2019)	Miura et al. (2019)	Perez-Garcia et al. (2020)	Haussen et al. (2020)	Renieri et al. (2022)	Meyer et al. (2022)	Okuda et al. (2022)	Baig et al. (2022)	Farouki et al. (2022)
**Combined Techniques**									
**Males (%)**	(41.4)*	15 (53)	27 (41)	16 (72)	109 (45.6)	-	(47.5)*	5 (71.43)	-
**Hypertension (%)**	(78.6)*	22 (78)	35 (66)	15 (68)	-	-	(67)*	3 (42.86)	-
**Dyslipidemia (%)**	(35.1)*	9 (32)	21 (39.6)	6 (27)	-	-	(37)*	3 (42.86)	-
**Diabetes Mellitus (%)**	(32.6)*	3 (10)	12 (22.6)	7 (32)	-	-	(19.5)*	1 (14.29)	-
**Prior Stroke (%)**	-	-	-	-	-	-	-	-	-
**Ischemic heart disease (%)**	(8.3)*	-	-	-	-	-	-	0 (0)	-
**Atrial fibrillation (%)**	(45.7)*	21 (75)	30 (56.6)	10 (45)	-	-	(69.5)*	3 (42.86)	-

**Smoker (%)**	-	()	17 (32.1)	()	69 (28.9)	-	-	-	-
**Single-Device Techniques**									
**Male (%)**	(41.7)*	18 (48)	24 (49)	77 (56)	49 (52.7)	-	(46.8)*	4 (28.57)	-
**Hypertension (%)**	(84.5)*	25 (67)	35 (71.4)	91 (66)	-	-	(64.4)*	8 (57.14)	-
**Dyslipidemia (%)**	(46.5)*	12 (32)	26 (53.1)	36 (26)	-	-	(29.9)*	4 (28.57)	-
**Diabetes mellitus (%)**	(25.7)*	5 (13)	13 (26.5)	34 (25)	-	-	(20.2)*	4 (28.57)	-
**Prior Stroke (%)**	-	-	-	-	-	-	-	-	-
**Ischemic heart disease (%)**	(8.6)*	-	-	-	-	-	-	2 (14.29)	-
**Atrial fibrillation (%)**	(49.3)*	23 (64)	28 (57.1)	41 (30)	-	-	(60.4)*	4 (28.57)	-
**Smoker (%)**	-	-	18 (36.7)	-	23 (24.7)	-	-	-	-

*Data not available for the DMVO-AIS subgroup of patients, percentages refer to the overall study population, which included anterior LVOs.

Figure S1. Forest plots comparing outcomes of combined techniques versus stent retriever alone in terms of: (A) mFPE (mTICI 2b-3); (B) successful final reperfusion (mTICI 2b-3); (C) complete final reperfusion (mTICI 2c-3); (D) functional independence (90-day mRS 0-2); (E) 90-day mortality; (F) symptomatic intracranial hemorrhage (sICH).

CI, confidence interval; FPE, first pass effect; mFPE, modified first pass effect; mRS, modified Rankin Scale; mTICI, modified Thrombolysis in Cerebral Infarction; OR, odds ratio; SR, stent retriever

(A)

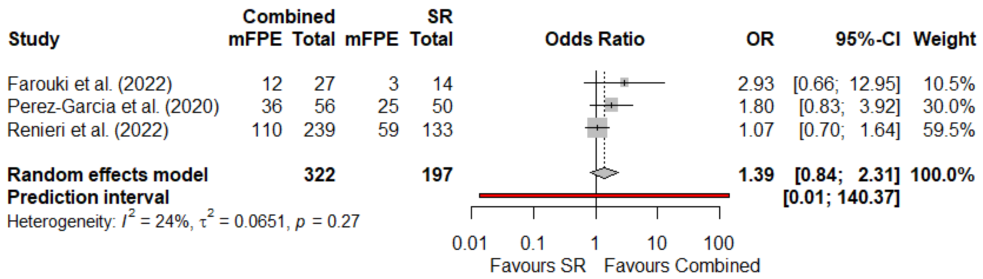

(B)

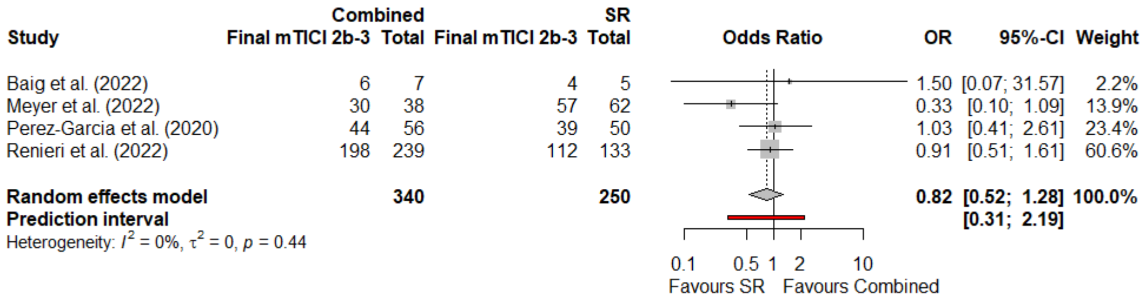

(C)

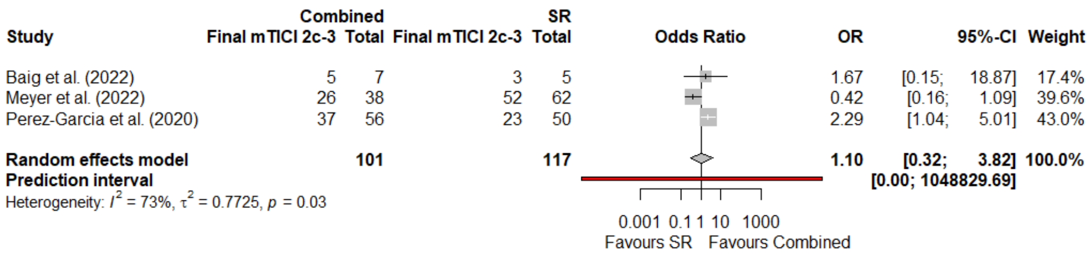

(D)

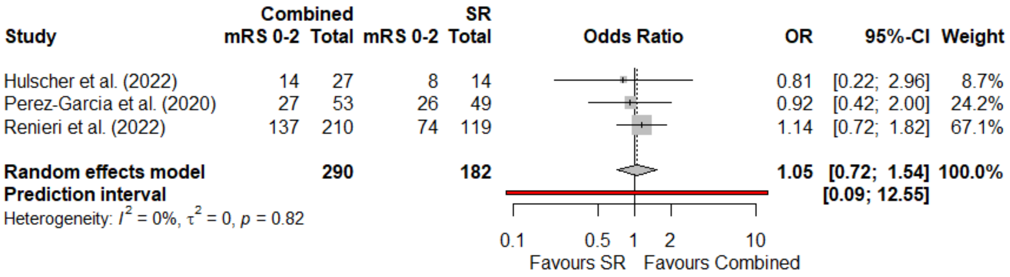
Study | Combined mRS 0-2 | Total | SR mRS 0-2 | Total | Odds Ratio | OR | 95%-CI | Weight
Hulscher et al. (2022) 14 27 8 14 0.81 [0.22; 2.96] 8.7%
Perez-Garcia et al. (2020) 27 53 26 49 0.92 [0.42; 2.00] 24.2%
Renieri et al. (2022) 137 210 74 119 1.14 [0.72; 1.82] 67.1%
Random effects model 290 182 1.05 [0.72; 1.54] 100.0%
Prediction interval [0.09; 12.55]
Heterogeneity: I^2 = 0%, τ^2 = 0, p = 0.82
0.1 0.5 1 2 10
Favours SR Favours Combined

(E)

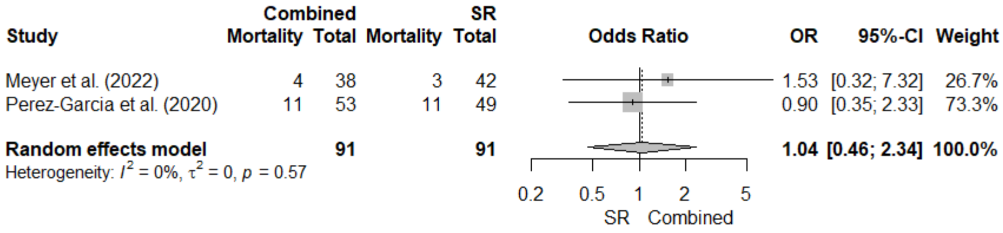
Study | Combined Mortality | Total | SR Mortality | Total | Odds Ratio | OR | 95%-CI | Weight
Meyer et al. (2022) 4 38 3 42 1.53 [0.32; 7.32] 26.7%
Perez-Garcia et al. (2020) 11 53 11 49 0.90 [0.35; 2.33] 73.3%
Random effects model 91 91 1.04 [0.46; 2.34] 100.0%
Heterogeneity: I^2 = 0%, τ^2 = 0, p = 0.57
0.2 0.5 1 2 5
SR Combined

(F)

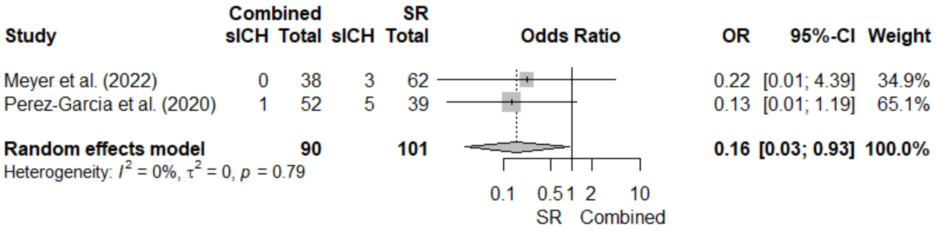
Study | Combined sICH | Total | SR sICH | Total | Odds Ratio | OR | 95%-CI | Weight
Meyer et al. (2022) 0 38 3 62 0.22 [0.01; 4.39] 34.9%
Perez-Garcia et al. (2020) 1 52 5 39 0.13 [0.01; 1.19] 65.1%
Random effects model 90 101 0.16 [0.03; 0.93] 100.0%
Heterogeneity: I^2 = 0%, τ^2 = 0, p = 0.79
0.1 0.5 1 2 10
SR Combined

Figure S2. Forest plots comparing outcomes of combined techniques versus direct aspiration alone in terms of: (A) mFPE (mTICI 2b-3); (B) successful final reperfusion (mTICI 2b-3); (C) complete final reperfusion (mTICI 2c-3); (D) functional independence (90-day mRS 0-2); (E) symptomatic intracranial hemorrhage (sICH).

CI, confidence interval; FPE, first pass effect; mFPE, modified first pass effect; mRS, modified Rankin Scale; mTICI, modified Thrombolysis in Cerebral Infarction; OR, odds ratio;SR, stent retriever

(A)

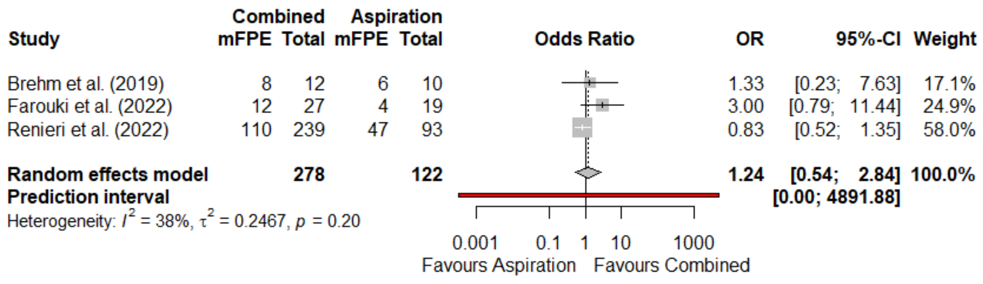

(B)

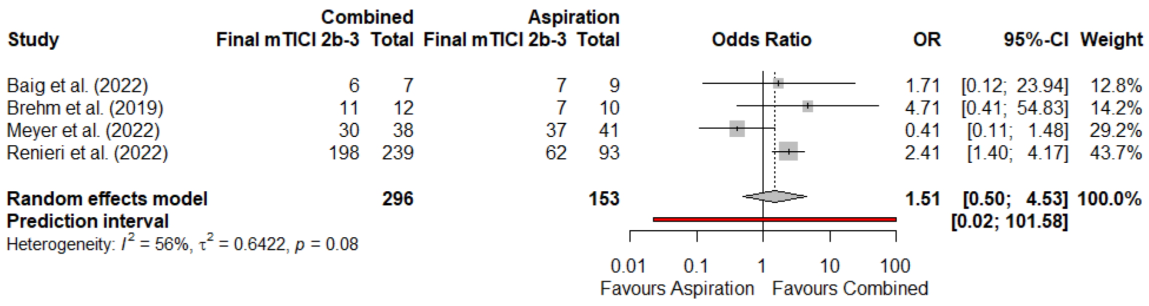

(C)

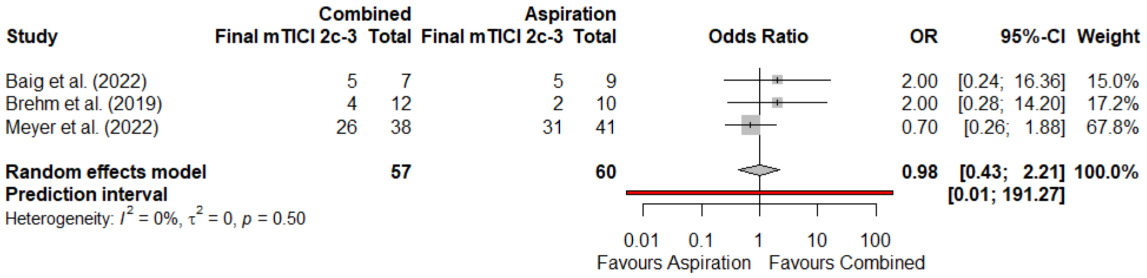
Combined
Aspiration
Study
Final mTICI 2c-3 Total Final mTICI 2c-3 Total
Odds Ratio
OR 95%-CI Weight
Baig et al. (2022) 5 7 5 9 2.00 [0.24; 16.36] 15.0%
Brehm et al. (2019) 4 12 2 10 2.00 [0.28; 14.20] 17.2%
Meyer et al. (2022) 26 38 31 41 0.70 [0.26; 1.88] 67.8%
Random effects model 57 60 0.98 [0.43; 2.21] 100.0%
Prediction interval [0.01; 191.27]
Heterogeneity: I^2 = 0%, τ^2 = 0, p = 0.50
0.01 0.1 1 10 100
Favours Aspiration Favours Combined

(D)

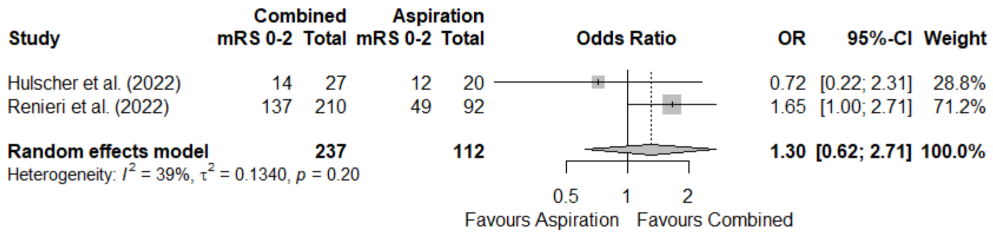
Combined
Aspiration
Study
mRS 0-2 Total mRS 0-2 Total
Odds Ratio
OR 95%-CI Weight
Hulscher et al. (2022) 14 27 12 20 0.72 [0.22; 2.31] 28.8%
Renieri et al. (2022) 137 210 49 92 1.65 [1.00; 2.71] 71.2%
Random effects model 237 112 1.30 [0.62; 2.71] 100.0%
Heterogeneity: I^2 = 39%, τ^2 = 0.1340, p = 0.20
0.5 1 2
Favours Aspiration Favours Combined

(E)

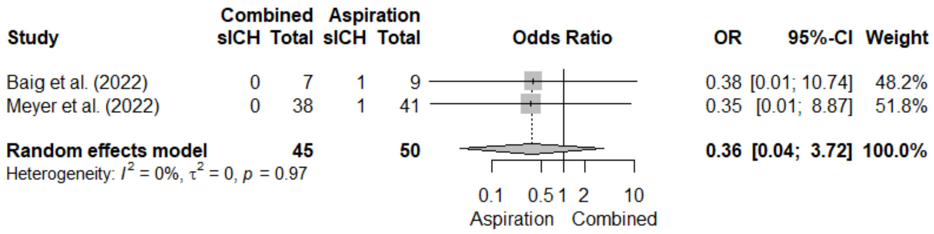
Combined
Aspiration
Study
sICH Total sICH Total
Odds Ratio
OR 95%-CI Weight
Baig et al. (2022) 0 7 1 9 0.38 [0.01; 10.74] 48.2%
Meyer et al. (2022) 0 38 1 41 0.35 [0.01; 8.87] 51.8%
Random effects model 45 50 0.36 [0.04; 3.72] 100.0%
Heterogeneity: I^2 = 0%, τ^2 = 0, p = 0.97
0.1 0.5 1 2 10
Aspiration Combined

Figure S3. Forest plots comparing technical outcomes of combined versus single-device techniques in terms of: (A) successful final reperfusion (mTICI 2b-3); (B) complete final reperfusion (mTICI 2c-3).

CI, confidence interval; mTICI, modified Thrombolysis in Cerebral Infarction; OR, odds ratio.

(A)

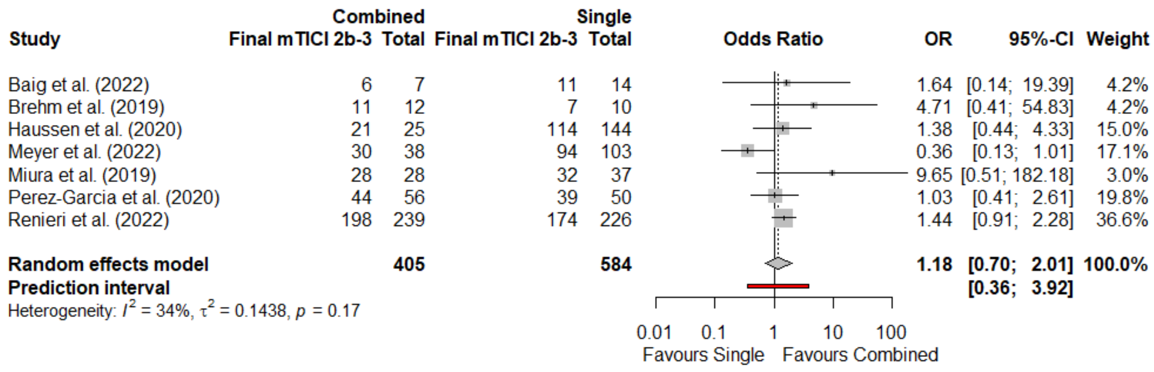

(B)

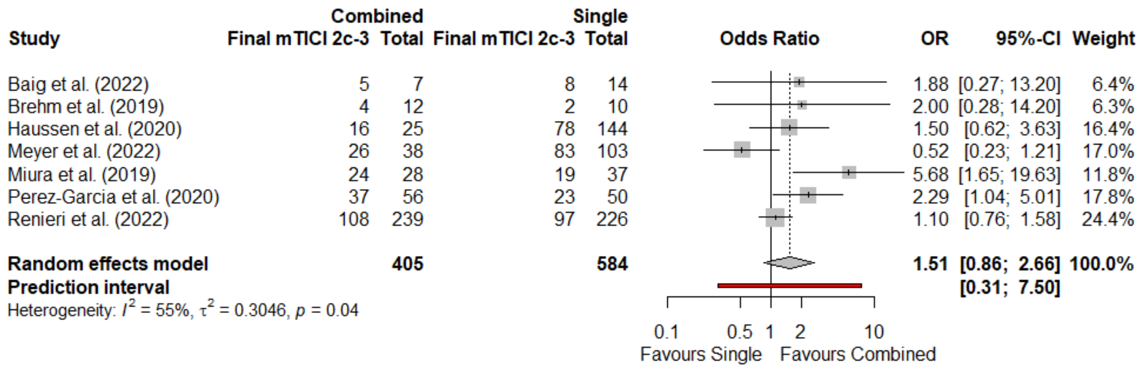

Figure S4. Forest plots comparing technical outcomes of combined versus single-device techniques with subgroup analysis by occlusion site, in terms of: (A) modified first-pass effect (mFPE); (B) first-pass effect (FPE); (C) successful final reperfusion (mTICI 2b-3); (D) complete final reperfusion (mTICI 2c-3)

CI, confidence interval; mTICI, modified Thrombolysis in Cerebral Infarction; OR, odds ratio.

(A)

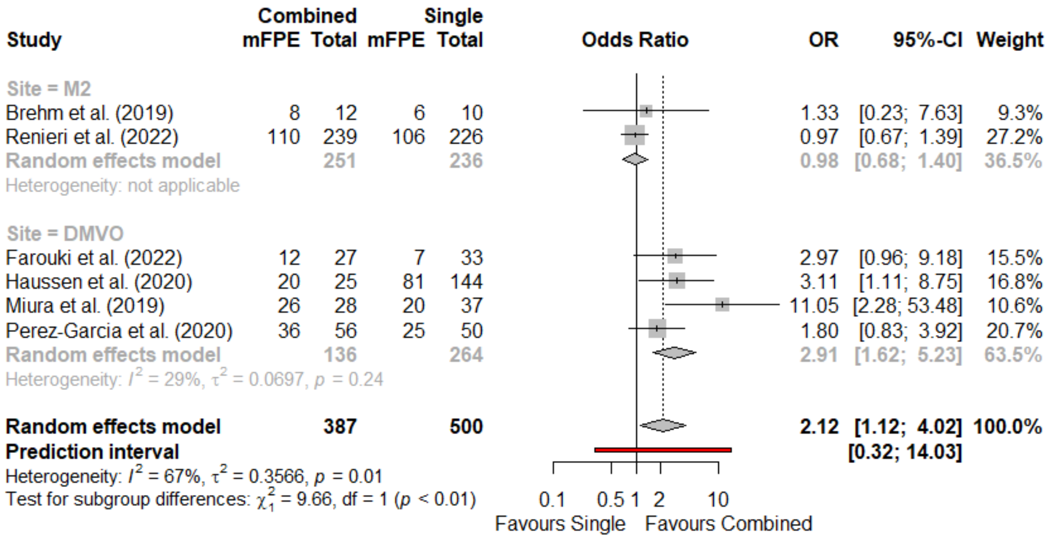

(B)

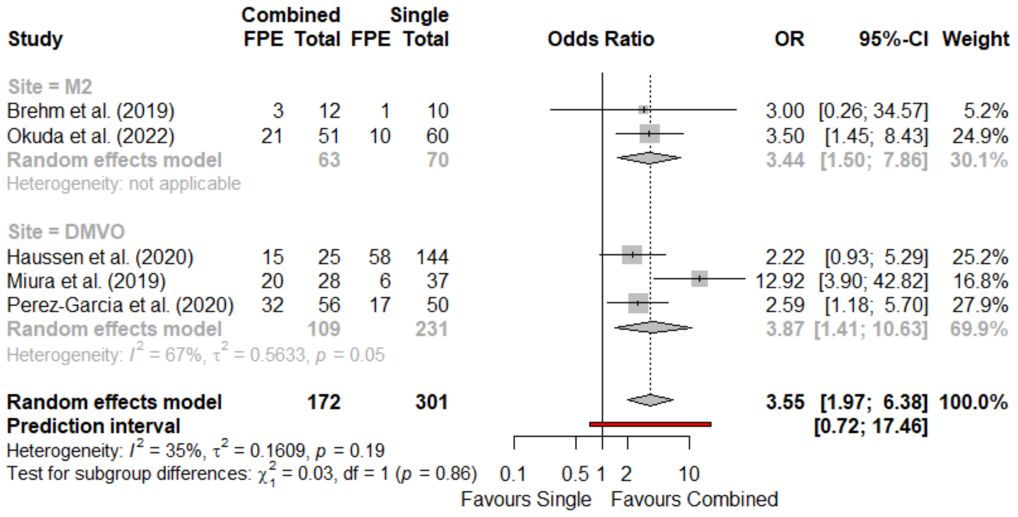

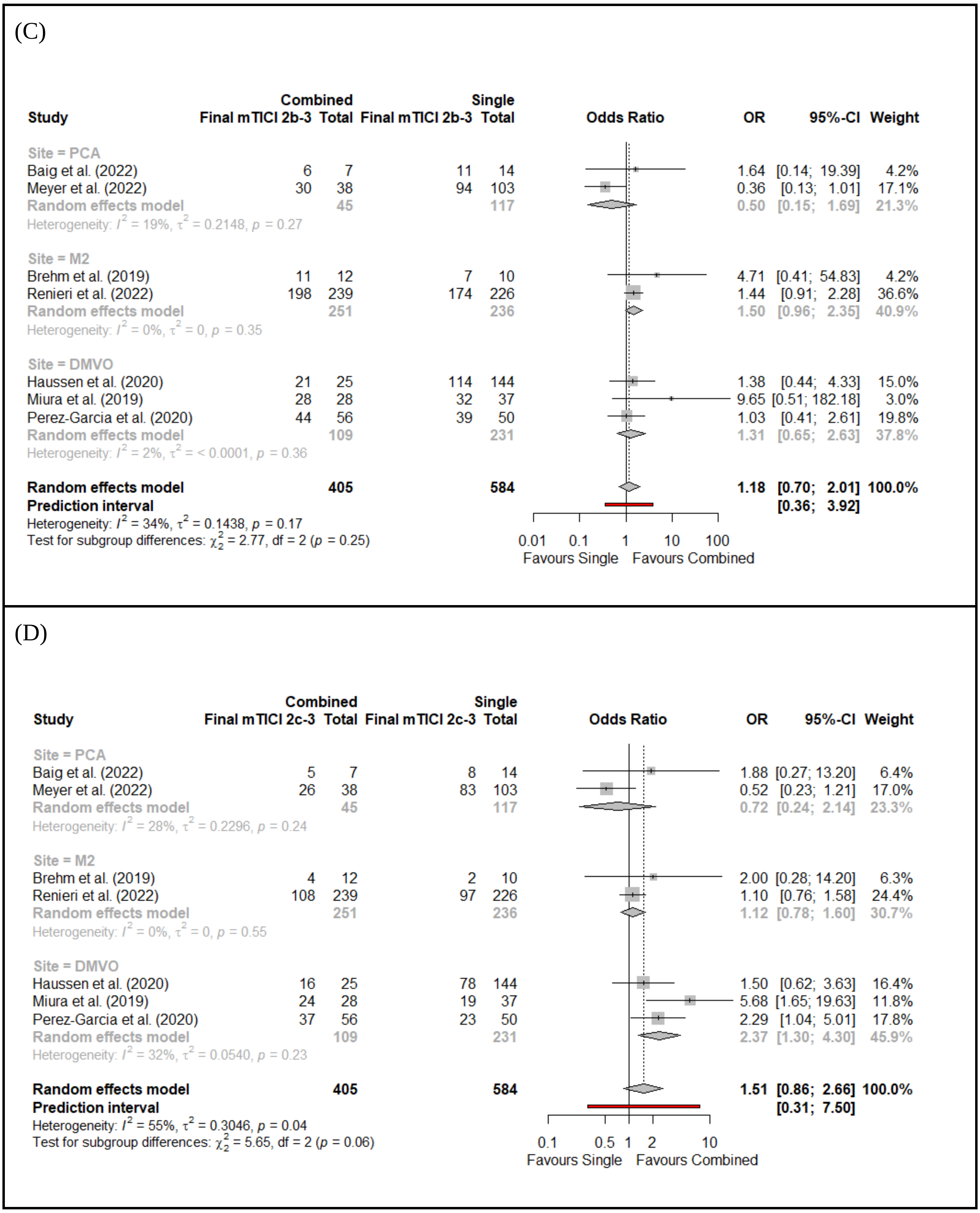
(C)
Study
Combined
Final mTICI 2b-3
Total
Single
Final mTICI 2b-3
Total
Odds Ratio
OR
95%-CI
Weight
Site = PCA
Baig et al. (2022) 6 7 11 14 1.64 [0.14; 19.39] 4.2%
Meyer et al. (2022) 30 38 94 103 0.36 [0.13; 1.01] 17.1%
Random effects model 45 117 0.50 [0.15; 1.69] 21.3%
Heterogeneity: I^2 = 19%, τ^2 = 0.2148, p = 0.27
Site = M2
Brehm et al. (2019) 11 12 7 10 4.71 [0.41; 54.83] 4.2%
Renieri et al. (2022) 198 239 174 226 1.44 [0.91; 2.28] 36.6%
Random effects model 251 236 1.50 [0.96; 2.35] 40.9%
Heterogeneity: I^2 = 0%, τ^2 = 0, p = 0.35
Site = DMVO
Haussen et al. (2020) 21 25 114 144 1.38 [0.44; 4.33] 15.0%
Miura et al. (2019) 28 28 32 37 9.65 [0.51; 182.18] 3.0%
Perez-Garcia et al. (2020) 44 56 39 50 1.03 [0.41; 2.61] 19.8%
Random effects model 109 231 1.31 [0.65; 2.63] 37.8%
Heterogeneity: I^2 = 2%, τ^2 = < 0.0001, p = 0.36
Random effects model 405 584 1.18 [0.70; 2.01] 100.0%
Prediction interval [0.36; 3.92]
Heterogeneity: I^2 = 34%, τ^2 = 0.1438, p = 0.17
Test for subgroup differences: χ^2_2 = 2.77, df = 2 (p = 0.25)
0.01 0.1 1 10 100
Favours Single Favours Combined
(D)
Study
Combined
Final mTICI 2c-3
Total
Single
Final mTICI 2c-3
Total
Odds Ratio
OR
95%-CI
Weight
Site = PCA
Baig et al. (2022) 5 7 8 14 1.88 [0.27; 13.20] 6.4%
Meyer et al. (2022) 26 38 83 103 0.52 [0.23; 1.21] 17.0%
Random effects model 45 117 0.72 [0.24; 2.14] 23.3%
Heterogeneity: I^2 = 28%, τ^2 = 0.2296, p = 0.24
Site = M2
Brehm et al. (2019) 4 12 2 10 2.00 [0.28; 14.20] 6.3%
Renieri et al. (2022) 108 239 97 226 1.10 [0.76; 1.58] 24.4%
Random effects model 251 236 1.12 [0.78; 1.60] 30.7%
Heterogeneity: I^2 = 0%, τ^2 = 0, p = 0.55
Site = DMVO
Haussen et al. (2020) 16 25 78 144 1.50 [0.62; 3.63] 16.4%
Miura et al. (2019) 24 28 19 37 5.68 [1.65; 19.63] 11.8%
Perez-Garcia et al. (2020) 37 56 23 50 2.29 [1.04; 5.01] 17.8%
Random effects model 109 231 2.37 [1.30; 4.30] 45.9%
Heterogeneity: I^2 = 32%, τ^2 = 0.0540, p = 0.23
Random effects model 405 584 1.51 [0.86; 2.66] 100.0%
Prediction interval [0.31; 7.50]
Heterogeneity: I^2 = 55%, τ^2 = 0.3046, p = 0.04
Test for subgroup differences: χ^2_2 = 5.65, df = 2 (p = 0.06)
0.1 0.5 1 2 10
Favours Single Favours Combined
